# EHMTI-0282. Deep brain stimulation in chronic cluster headache: lead location, clinical response and neuronal signatures

**DOI:** 10.1186/1129-2377-15-S1-E19

**Published:** 2014-09-18

**Authors:** S Miller, F Rasul, M Matharu, A Pogosyan, P Brown, M Hariz, L Zrinzo

**Affiliations:** 1 Headache, UCL Institute of Neurology, London, UK; 2 Functional Neurosurgery, UCL Institute of Neurology, London, UK; 3 Nuffield Department of Clinical Neurosciences, University of Oxford, Oxford, UK

## Background

Chronic Cluster Headache (CCH) is refractory to medical therapy in a small minority of patients. Neuroimaging implicateS the ipsilateral posterior hypothalamic region (PH) in its pathogenesis. PH deep brain stimulation (PH-DBS) has shown promise in the management of refractory CCH. Here we investigate the value of local field potentials (LFPs) in relation to the site of active DBS contact used for chronic stimulation.

## Methods

Four patients with refractory CCH treated with PH-DBS were investigated. The target on stereotactic T2-weighted images lay in the anteromedial quadrant between the red nucleus and mammillary bodies. LFPs were recorded from externalised wires prior to implantation of the impulse generator a week after lead implantation.

## Results

All leads were within 1.0mm of the intended target point. Mean(range) coordinates of the chronically active contact were 3.6(2.0-5.40)mm lateral, 3.1(1.2-5.0)mm posterior and 5.0(2.0-8.6)mm inferior to the midcommissural point. Mean follow-up was 23(15-26) months. Headache load was defined as the [severity (on the visual analogue scale)] x [duration] x [frequency]) of cluster headache. Headache load reduced by 57, 58, 68 and 100% in each patient. LFP peaks were noted in 3 out of 4 cases (at 8, 11 and 15Hz). The contacts used for stimulation at last follow-up coincided with one or both of the contacts affording the highest amplitude peak in bipolar recordings.

## Conclusions

Our results support previous data that PH-DBS may be useful in patients with medically refractory cluster headache. LFP activity may potentially be useful in guiding contact selection during programming and deserves further investigation.

No conflict of interest.

